# Translating PrEP effectiveness into public health impact: key considerations for decision-makers on cost-effectiveness, price, regulatory issues, distributive justice and advocacy for access

**DOI:** 10.7448/IAS.18.4.19973

**Published:** 2015-07-20

**Authors:** Catherine Hankins, Ruth Macklin, Mitchell Warren

**Affiliations:** 1 Department of Global Health, Academic Medical Center, Amsterdam Institute for Global Health and Development, University of Amsterdam, Amsterdam, The Netherlands; 2 Department of Infectious Disease Epidemiology, Faculty of Epidemiology and Population Health, London School of Hygiene and Tropical Medicine, London, England; 3 Epidemiology & Population Health, Albert Einstein College of Medicine New York, NY, USA; 4 AVAC New York, NY, USA

**Keywords:** HIV prevention, pre-exposure prophylaxis, ethics, cost-effectiveness, regulatory, antiretroviral, tenofovir/emtricitabine, resource allocation

## Abstract

**Introduction:**

The extraordinary feat of proving the effectiveness of oral pre-exposure prophylaxis (PrEP) in clinical trials in different populations in a variety of settings may prove to have been easier than ensuring it is used well. Decision-makers must make difficult choices to realize the promise of antiretroviral prophylaxis for their countries. This paper outlines key economic, regulatory and distributive justice issues that must be addressed for effective and acceptable PrEP implementation.

**Discussion:**

In considering the role that PrEP can play in combination prevention programmes, decision-makers must determine who can benefit most from PrEP, how PrEP can be provided safely and efficiently, and what kind of health system support will ensure successful implementation. To do this, they need contextualized information on disease burden by population, analyses of how PrEP services might best be delivered, and projections of the human resource and infrastructure requirements for each potential delivery model. There are cost considerations, varying cost-effectiveness results and regulatory challenges. The principles of ethics can inform thorny discussions about who should be prioritized for oral PrEP and how best to introduce it fairly. We describe the cost-effectiveness of PrEP in different populations at higher risk of HIV exposure, its price in low- and middle-income countries, and the current regulatory situation. We explore the principles of ethics that can inform resource allocation decision-making about PrEP anchored in distributive justice, at a time when universal access to antiretroviral treatment remains to be assured. We then highlight the role of advocacy in moving the PrEP agenda forward.

**Conclusions:**

The time is ripe now for decisions about whether, how and for whom PrEP should be introduced into a country's HIV response. It has the potential to contribute significantly to high impact HIV prevention if it is tailored to those who can most benefit from it and if current regulatory and pricing barriers can be overcome. Advocacy at all levels can help inform decision-making and push the access agenda to avert HIV infections among those at highest risk of HIV exposure. The benefits will accrue beyond the individual level to slow HIV transmission at the population level.

## Introduction

In clinical trials conducted in Africa, Asia, Europe and North America over the past decade, pre-exposure prophylaxis (PrEP), using oral formulations of the antiretroviral drugs tenofovir or tenofovir/emtricitabine (TDF/FTC), has been shown to significantly reduce the risk of HIV acquisition among men who have sex with men (MSM) [1–3], heterosexual men and women [4, 5], and people who inject drugs (PWID) [6]. Trials involving monthly insertion of a vaginal ring containing the antiretroviral dapivirine will report results in 2016 [7, 8]. Following good safety signals [9], long-acting PrEP is currently being investigated in trials of injectable rilpivirine [10] and cabotegravir [11].

With vaginal rings and injectables potentially coming on the heels of proven oral PrEP, it is important to consider how best to introduce these new options into current HIV combination prevention strategies [12, 13] to achieve reductions in HIV risk at individual and community levels. Thus far, a daily oral PrEP product, TDF/FTC (Truvada^®^), has been approved for use but only in the United States [14]. This US Food and Drug Administration (FDA) regulatory approval in July 2012 was quickly followed by initial World Health Organization (WHO) guidelines for the conduct of PrEP demonstration projects [15]. More recently, WHO has issued additional guidance for key populations [16].

A large number of demonstration projects are now underway in trial-naïve populations [17], complementing the post-trial access studies among participants of the original trials reporting efficacy. In addition, the Partners PrEP trial randomized participants in the former placebo arm to either TDF/FTC or tenofovir alone, since both products reduced HIV acquisition risk, and found that the lower-cost single drug also provides high protection [18]. This suite of projects and studies is providing valuable data to inform country implementation strategies.

Decision-makers, faced with the results of a plethora of studies in diverse populations and regulatory approval for oral PrEP only in the United States, have difficult choices to make to realize the promise of antiretroviral prophylaxis [19] for their countries. They need contextualized information on disease burden by population, analyses of how PrEP services might best be delivered, and assessment of the human resource and infrastructure requirements for potential delivery models. There are cost considerations, varying cost-effectiveness results, regulatory challenges and ethical concerns that must be addressed to ensure that oral PrEP is a tangible HIV prevention choice for those individuals who can most benefit from it. Complementing other papers in this supplement addressing PrEP, this paper outlines key economic, regulatory, distributive justice, and access issues that must be addressed in each context to realize the full potential of effective and acceptable PrEP implementation.

## Discussion

### Cost-effectiveness, pricing and trade-offs

#### Cost-effectiveness

Cost-effectiveness analysis is a standard method used for allocating resources in health policy. It seeks to determine how the most effective policy can be implemented at the least cost. Studies of oral PrEP cost-effectiveness have used a variety of metrics, estimating cost per HIV infection averted [20–22], cost per quality-adjusted life year (QALY) gained [21, 23–27], cost per disability-adjusted life year (DALY) averted [28], cost per year life saved [29] and PrEP years per infection averted [30]. The studies have examined PrEP for heterosexual transmission in southern Africa [20] and South Africa [21, 22, 29, 31–33] and for other modes of transmission, among PWID in Ukraine [25] and MSM in the USA [23, 24, 26, 27] and Peru [28].

A systematic review of 13 cost-effectiveness studies found that key considerations to address in assessing cost-effectiveness of PrEP are cost, epidemic context, individual adherence level, PrEP programme coverage and prioritization strategy [34]. PrEP could be a potentially cost-effective addition to HIV-prevention programmes, particularly when those at highest risk of HIV exposure are prioritized, although drug costs would limit cost-effectiveness. While PrEP could have impact in key populations such as MSM, the first priority for PWID might be expanding access to antiretroviral treatment (ART) and opioid substitution therapy. In considering trade-offs, prioritizing PrEP for young women in southern Africa who are at alarmingly high risk of HIV acquisition can be cost-effective, especially when there are costly obstacles to recruiting HIV-positive people for treatment using the same drug [35].

Cost-effectiveness studies guide resource allocation decisions by indicating where resources can be applied for greatest impact. Funding PrEP while other potentially more cost-effective HIV prevention interventions remain under-funded may have high opportunity costs, diverting resources from early ART initiation or other prevention strategies [34]. It is therefore important, as oral PrEP moves into demonstration projects and regular use in some settings, to obtain and integrate real-world costing data for all PrEP programme elements to replace earlier hypothetical costs. This will assist policy-makers in planning future resource allocations for PrEP as part of high-impact combination prevention.

#### Pricing

The price of drugs is a key component of overall programme costing. The price of tenofovir-containing ART regimens from originator sources has remained static since 2007, while the lowest prices of stand-alone tenofovir fell by almost half in 12 months, from $48 per person year in 2013 to $26 in 2014 [36]. In 2012, it was estimated that over half of all people on ART in countries with generic access were on tenofovir-based regimens, with this proportion estimated to rise to 70% of patients on first-line treatment regimens by the end of 2014 [36].

#### Trade-offs

Paying for tenofovir-based PrEP when access to ART is not universal is an issue that requires careful reflection in each context. People who do not acquire HIV because of the effective use of PrEP when they are at most risk of HIV acquisition will avoid lifelong ART and its associated costs.

Drugs that are not used for ART, such as maraviroc, which is currently being assessed in the Next-PrEP clinical trial [37], would not present direct competition for drug use, but the overall issue of resource allocation remains. The dapivirine vaginal ring, replaced monthly, is being assessed in two Phase III trials [7, 8]. Phase I trials of the long-acting injectables, bi-monthly rilpivirine (TMC 278), a non-nuclease reverse transcriptase inhibitor [38], and tri-monthly cabotegravir (S/GSK1265744), an integrase inhibitor [39], have reported safety and tolerability [9] with Phase II trials following suit [10, 11]. Thus, the potential array of delivery options could expand to provide choices for PrEP that would not necessarily compete with treatment drug demands. Issues of cost, access, equity and trade-offs will remain. However, without regulatory approval no PrEP option can be rolled out where it is most needed.

### Regulatory issues

**Figure 1:**
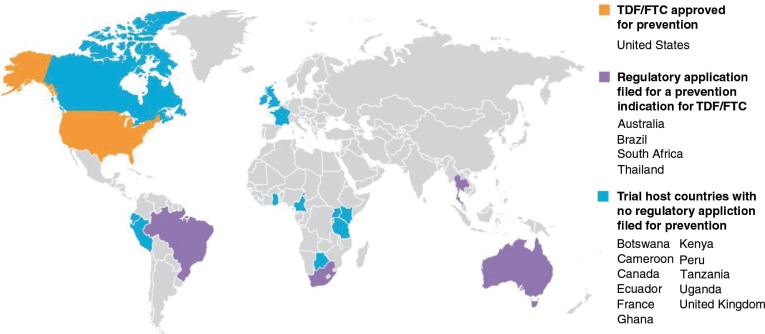
Regulatory approval in trial host countries for daily TFD/FTC. Permission to use granted by AVAC.

Following FDA approval of oral TDF/FTC for a prevention indication, its manufacturer Gilead Sciences, Inc. applied for approval in 4 of the 14 countries that hosted TDF/FTC PrEP trials: South Africa (December 2013), Thailand (April 2014), and Australia and Brazil (late 2014). Figure 1 outlines the countries in which PrEP trials have taken place and the current status of regulatory review for a prevention indication of oral TDF/FTC [40].

It is unclear when the four pending applications will be decided and when the company might apply in the other host trial countries. The drug has been registered for HIV treatment in 154 countries worldwide, including 110 low- and middle-income countries [41]. It is available as Gilead-branded Truvada^®^ or as generic versions in developing countries through Gilead's partnerships with generic manufacturers. Gilead Sciences was the first pharmaceutical company to commit to the Medicines Patent Pool, a United Nations-backed organization established in 2010 to improve access to appropriate, affordable HIV medicines and technologies for people living with HIV in developing countries, and recently signed a licence for the new medicine tenofovir alafenamide (TAF) [42].

Thus, while TDF/FTC is already available in many countries as an approved therapeutic, the absence of a prevention indication outside the United States limits programmatic and policy decisions to expand access to PrEP. However, recent clinical trial developments may move TDF/FTC onto a faster track for regulatory approval. The PROUD trial of daily oral PrEP in England was unblinded in October 2014 on the recommendation of its Data Safety Monitoring Board when it became no longer ethical, in light of the compelling findings, to continue the delayed arm that had no access for 12 months [43]. Two weeks later, the IPERGAY trial of event-driven PrEP (2 pills before sex and 1 pill 24 h and 48 h later) in France and Canada was unblinded and its placebo arm offered PrEP [44]. PROUD found that PrEP reduced HIV risk by 86% (90% CI: 58;98) compared with no-PrEP (*p*=0.0002) [2], while Ipergay also reported an 86% reduction in HIV incidence (95% CI: 40;99), *p*=0.002 [3]. Gilead Sciences is considering submission to the European Medicines Agency (EMA) for regulatory approval, which would expand access in Europe. However, EMA regulatory approval for low- and middle-income countries can only be done for medicinal products for human use that are intended exclusively for markets outside the European Union [45].

## Ethical considerations

### Principles of distributive justice

While cost-effectiveness analysis seeks to determine how the most effective policy can be implemented at the least cost, the result may conflict with the application of ethical principles designed to introduce important values other than monetary ones. Examining leading principles of justice can identify different priorities for allocation of PrEP although the principles can conflict, requiring a balance of competing concerns. There is no uniquely correct way of doing this balancing. Furthermore, there is no consensus on what weight to give to the different principles [46]. It has even been argued that the impossibility of achieving a consensus on which principle to choose requires abandoning the search for substantive principles of justice and instead, introducing a method that involves fairness in the procedural aspects of allocation decision-making [47]. However, procedural fairness does not guarantee fairness as an outcome that would accord with any of the leading substantive principles of distributive justice.

The principle of **utility** [48, 49] is the one most widely used in health policy: choose the option that has the most beneficial consequences and the fewest harmful consequences for society as a whole. The philosophical and economic literature contains numerous versions of utilitarian theory. A general form of consequentialist utility theory, known as Total Consequentialism, indicates that “… moral rightness depends only on the *total* net good in the consequences (as opposed to the average net good per person)” [50]. Applying this principle requires specifying which consequences are to count: minimizing costs, preventing new infections or ensuring fairness in the distribution of PrEP. It is evident that the utilitarian principle can yield different results depending on an array of empirical facts and circumstances.

Two principles that are sometimes conflated are the **egalitarian** principle [49] and the principle of **equity** [51, 52]. Whereas the egalitarian principle mandates treating all in need equally, the principle of equity allows for contextual factors to be considered in a fair distribution of resources. When deciding which groups should receive PrEP first as prevention programmes are scaled up, decision-makers should consider whether all in need should be treated equally or whether certain groups, such as those who are marginalized, stigmatized or typically underserved, should be given preference. A well-known application of the **egalitarian** principle is a lottery among the pool of potential users when supplies are limited. Egalitarian principles make no distinctions regarding who might benefit most from an intervention or what choice would best serve the goals of public health. The principle of **equity** has a specific meaning in the context of access to health care: “The dominant conceptualization of equitable access to health care among health service researchers builds on the idea that the utilization of services should reflect actual needs for care” [53]. Application of this principle to PrEP might focus on traditionally underserved populations, as well as on young women at high risk of infection because poverty, misogyny and their limited social capital make them less able than others to avoid unprotected sex.

The **prioritarian** principle [54] calls for ensuring that resources are provided to the least-advantaged members or groups in society. In the context of HIV prevention, these might be those at greatest risk of becoming infected, the poorest people, the most vulnerable or the most highly stigmatized.

It is clear that applying principles of distributive justice for making health care allocations cannot guarantee justice in outcomes. They are designed for selecting populations to be given priority in a particular context, with the principle of equity designed to eliminate socio-economic and other barriers to care.

### Allocating resources for HIV prevention

Because resources for PrEP are insufficient to meet the needs of all who could benefit, decisions are needed about which populations should be given priority for receiving PrEP. People who engage in behaviour that places them at higher risk of HIV acquisition and whose sexual networks likely extend beyond their own subpopulation are an obvious choice because they have the greatest likelihood of transmitting HIV if they acquire it. Providing them with access to antiretroviral prevention first may mean that HIV infection will spread more slowly in a country. Thus, a logical choice as early priority populations for receiving PrEP could be young women, sex workers, MSM and PWID; however, these may be among the hardest people to reach.

The **prioritarian** principle operates as a constraint on the utilitarian principle to ensure that the most disadvantaged individuals are not ignored in the effort to scale up HIV prevention [46]. Different criteria exist for determining who are the most disadvantaged. Populations already identified as early priorities for HIV prevention according to the utilitarian principle appear also to be among the least advantaged according to several criteria. In most societies, they are stigmatized, marginalized, and typically engaged in illegal behaviour, and are often at greater risk from authorities. This includes PWID in countries with punitive drug policies, MSM in some African countries where homosexual behaviour is a criminal offense and sex workers in most countries worldwide. Thus, the utilitarian and prioritarian principles concur that those who are most at risk of HIV exposure should be the first to receive PrEP.

The fastest growing group of newly infected people in sub-Saharan Africa is young women. According to UNAIDS, “In sub-Saharan Africa, women and girls account for almost 57% of adults living with HIV. Recent surveys reveal that in South Africa, Zambia, and Zimbabwe, young women (age 15–24) are five to six times more likely to be infected than young men of the same age” [55]. The principle of **equity** might, therefore, call for scaling up HIV testing and offering PrEP to young HIV-negative women who are likely to be at risk.

Finally, HIV serodiscordant couples might be prioritized for PrEP, when the HIV-positive partner, whether on ART or not, is not virally supressed. A 96% reduction in HIV-negative partners’ risk of HIV acquisition was recently reported in a study of HIV-serodiscordant couples offered immediate ART for the HIV-positive partner and PrEP for the HIV-negative partner [56]. According to the principle of **urgent need** [57], HIV-negative partners in serodiscordant couples where the HIV-positive partner is not virally suppressed would have priority for PrEP because they risk acquiring HIV with each sexual act.

The urgent need principle can be combined with the utilitarian principle in setting priorities for allocating PrEP, with the principle of equity giving priority to stigmatized and marginalized populations, such as MSM, sex workers and PWID, and young women and serodiscordant couples.

## Advocacy for access

Since the early PrEP trial controversies [58, 59], advocates have played an active role in monitoring trials, interpreting trial results, advocating for access, disseminating information and, in the United States, engaging in the regulatory process.

Shortly after several of the first oral PrEP trials were stopped in 2005 amidst controversy, AVAC and UNAIDS began a consultative process with civil society representatives, researchers and funders to develop approaches to guide productive engagement in research. The resulting Good Participatory Practice Guidelines [60] are now increasingly used across a range of HIV prevention trials and have been adapted for use in non-HIV research efforts [61].

Following the iPrEx trial results in 2010 [1], a number of advocacy groups played leading roles in their countries and communities to explain clinical trial results and push for evidence-based policies and programmes. A coalition of 14 US HIV and health advocacy organizations submitted extensive public comments to the FDA in 2012 to support approval of TDF/FTC as PrEP. Their written comments, as well as formal presentations at the public FDA Advisory Committee meeting, pointed to the compelling evidence on the efficacy of PrEP and highlighted the unique potential of this intervention [62].

Advocacy groups, representing diverse populations who need and could most benefit from PrEP, especially in countries where the PrEP trials took place, also called for an ambitious, well-coordinated scale-up of demonstration projects across diverse populations of men, women and transgender people at risk for HIV through sex [63, 64]. For example, the US Women and PrEP Working Group, a coalition of women from leading AIDS and women's health organizations, advocates for a national agenda to answer questions about the best way to make PrEP available to women as a prevention option.

Finally, web-based community efforts include AVAC's PrEP Watch website (www.prepwatch.org), a clearinghouse for information on PrEP, and the AIDS Foundation of Chicago's My PrEP Experience (www.myprepexperience.blogspot.com), featuring stories from people who have chosen to use PrEP.

## Conclusions

Decision-makers considering the introduction of PrEP in their countries are faced with competing priorities and the need to address key economic, regulatory, distributive justice and access issues. Unless these processes are informed by inputs relative to their own specific context, it will be difficult to realize the full potential of effective and acceptable PrEP implementation. Using disease burden, costing information and known effectiveness, cost-effectiveness studies that illustrate the utilitarian principle at work can provide an initial indication of the potential impact of PrEP programmes. The results may conflict with the application of egalitarian or prioritarian principles of distributive justice.

The price of PrEP varies widely and the US FDA is the only regulatory agency to approve it for HIV prevention thus far. This approval has helped pave the way for greater access and insurance reimbursement for PrEP in the United States, but even there access challenges remain for some seeking PrEP. Although the regulatory pathway in Europe seems clearer in light of the recent European trial results, answers will be needed to the question of who will pay. Countries that lack regulatory capacity to independently evaluate the use of TDF/FTC as PrEP might use WHO pre-qualification and guidance, when available. The question of who will pay for PrEP in low- and middle-income countries requires frank discussions at national level and with international donors supporting strategies to end AIDS. These will need to be underpinned by discussions of equity and ethical allocation under conditions of limited resources.

Transitioning from clinical trial efficacy to public health impact is never easy. Experiences with preventing vertical transmission, programming the female condom, providing post-exposure prophylaxis (PEP), delivering sterile needles and scaling up voluntary medical male circumcision, amongst others, provide cogent examples in HIV prevention of slow policy and programme responses, unrealized expectations and resultant limited impact. There are important lessons to be learned about the factors that facilitate and impede uptake of new HIV prevention innovations [65].

PrEP is not meant for everyone, all of the time. If done well, though, initial PrEP introduction activities will enable policy-makers and programme planners to answer the questions of who can benefit most from PrEP, how to provide it safely and efficiently, how to integrate PrEP into combination treatment and prevention programmes, and what kind of health system support is needed to ensure successful implementation.

The extraordinary feat of proving PrEP's efficacy may turn out to have been easier than ensuring that it is used well. This is not unique to PrEP and insights can be gleaned from experiences with implementation of other novel strategies. Ensuring that PrEP fulfils its potential as part of high-impact combination HIV prevention requires establishing the additional evidence, education, support services and resources that are needed, as well as the regulatory framework and cost scenarios for access to PrEP.
